# Investigating the loss of work productivity due to symptomatic leiomyoma

**DOI:** 10.1371/journal.pone.0197958

**Published:** 2018-06-11

**Authors:** Klara Hasselrot, Mia Lindeberg, Peter Konings, Helena Kopp Kallner

**Affiliations:** 1 Department of Obstetrics and Gynecology, Danderyd Hospital, Stockholm, Sweden; 2 Department of Clinical Sciences at Danderyd Hospital, Division of Obstetrics and Gynecology, Karolinska Institute, Stockholm, Sweden; 3 Gedeon Richter Nordics AB, Stockholm, Sweden; 4 Parexel International, Stockholm, Sweden; Indiana University School of Medicine, UNITED STATES

## Abstract

**Introduction:**

Leiomyoma affects up to 50% of fertile women, leading to morbidity such as bleeding or pain. The effect of symptomatic leiomyoma on the productivity of employed women is understudied. The present study investigates productivity loss in a Swedish setting in women with symptomatic leiomyoma compared to healthy women.

**Material and methods:**

Women seeking care for leiomyoma and heavy menstrual bleeding (HMB) were recruited at nine Swedish sites. Healthy controls with self-perceived mild to normal menstruation were recruited at routine visits. Cases and controls were employed without option to work from home. After recruitment, all women reported the work productivity and activity impairment (WPAI) questionnaire, the pictorial blood assessment chart (PBAC) and pain on the visual analog scale (VAS).

**Results:**

Women with symptomatic leiomyoma (n = 88) missed more working time during menses compared to asymptomatic controls (n = 34): 7.6 vs 0.2% p = 0.003. The proportion of impairment while working was also significantly higher in women with symptomatic leiomyoma (43.8 vs 12.1% p<0.001). Moreover, cases reported greater activity impairment outside office hours (43.9 vs 12.1%, p<0.001). Among healthy controls, 69.5% reported symptoms of HMB (PBAC>100).

**Conclusions:**

Symptomatic leiomyoma leads to loss of working hours as well as loss of productivity during working hours, and affects women in other daily activities. Increased awareness of the impact of leiomyomas on women's lives is needed, and timely and appropriate management of the symptomatic leiomyomas could improve work productivity and quality of life.

## Introduction

Leiomyoma (or uterine fibroids) are the most common benign pelvic tumor in women, reaching lifetime incidences of up to 77% [1], and can be found via imaging in 50% of reproductive-age women at any given time [2]. Although up to 50% of leiomyomas are estimated to be asymptomatic, the main reasons to seek medical care are heavy menstrual bleeding (HMB), pelvic pain, pressure symptoms or fertility disorders [3]. The symptomatology is clinically relevant foremost in menstruating women, potentially causing difficulties during working hours as well as in daily life. Symptomatology due to leiomyoma increases with age until menopause, when the tumors most commonly stop proliferating and symptoms usually decline or disappear[4]. Treatment options for symptomatic leiomyoma are several and varying, ranging from over the counter iron supplements to complicated abdominal surgery. Treatment modality depends on the overall burden as well as the location of the tumors in the uterus. New treatments are continuously emerging through pharmacological agents as well as developed surgical procedures, although fertility sparing regimens are still in minority. Regardless of menopausal status, leiomyoma is the leading cause of hysterectomy in the world [5].

The clinical definition of HMB is a menstrual blood loss (MBL) of >80mL per cycle [6]. A valid screening instrument for patient suffering from HMB, with a sensitivity of >80%, is the pictorial blood assessment chart (PBAC) with a cut-off of 100[7]. Later studies by Zakerhah et al confirm the use of PBAC as a diagnostic tool of HMB, however different cutoffs for heavy menstrual bleeding with PBAC have been proposed [8].

Since menstruating women are often family providers, cyclic symptoms keeping them from full professional capacity may have a great impact on productivity, both in personal and professional settings. The sum of direct costs (such as surgery or medication) and indirect costs (such as work productivity loss) have recently been reviewed by Soliman et al in the United States. Costs were estimated to range from USD 11 000 to 25 000 per patient per year after diagnosis and/or surgery[9]. The authors highlight a striking discrepancy between the United States and the rest of the world regarding this research field, where 19 of 26 studies on productivity loss emanate from the US and only five from Europe. Furthermore, most research focuses on direct costs such as comparing different treatment strategies, or investigating the costs from an employer’s perspective, while the patient’s perspective is often absent [9]. To our knowledge there are no studies investigating whether leiomyoma is affecting working women in a Nordic setting. We therefore sought to investigate work productivity loss due to symptomatic leiomyoma in a multicenter study at nine Swedish sites.

## Material and methods

All nine centers participating in the study provide specialized gynecological care, and although referral is not mandatory, many patients are being referred from general practitioners, outpatient gynecologists or midwives. Cases were recruited from patients seeking a gynecologist due to symptoms related to leiomyoma. Controls were recruited at routine visits for various other reasons such as cytology cervix cancer screening. Inclusion criteria for cases were: 1) known leiomyoma and 2) self-perceived heavy menstrual bleeding. For controls, the inclusion criteria were 1) self-perceived mild to normal menstrual bleeding and menstrual pain. For both groups, the following inclusion criteria applied: 1) 30–55 years of age, 2) currently employed without option to work from home, 3) sufficient knowledge of the Swedish language.

Each woman participating in the study signed a written informed consent form after oral and written information had been provided. Once included, each participant was further given a code, and all demographic information related to the patient were stored separately at each respective site, and was only used to send out reminders about study completion. All study subjects could withdraw their participation at any given time at their own request.

Women received compensation of 20 Euro in the form of a gift card after they had filled in the online questionnaire.

The study was approved by the institutional review board at Karolinska Institutet with number 2014/1524-31.

To measure work loss and menstrual symptoms—bleeding pattern as well as pain—in both groups, we relied on the validated work productivity and activity impairment (WPAI) questionnaire, the pictorial blood assessment chart (PBAC, range 0-∞) for menstrual blood loss and the visual analogue scale (VAS, range 0–100) for assessment of pain [7, 10, 11]. All parameters were evaluated during the first menstrual period post enrollment. In addition, women also recorded their burden of illness due to menstrual symptoms in a second questionnaire, where they were asked if the menstruation limited them in different work-related or social situations. At inclusion, all women were provided with a link to the online versions of the questionnaires, as well as a paper version along with a pre-paid envelope, to complete during and after the first menstrual period post enrollment. During the investigated menstrual period, no new treatment for symptoms of leiomyomas was allowed. The investigator on site secured date of birth and contact information for each participant.

The primary endpoint of this study was to investigate the difference in days of work productivity between women with symptomatic uterine fibroids compared to asymptomatic women (cases and controls), measured as the percent work time missed due to symptoms related to menstruation (WPAI 1 = absenteeism). Secondary endpoints were: the difference in impairment measured as the percent impairment while working due to symptoms related to menstruation (WPAI 2 = presenteeism), the difference in overall work impairment measured as the percent overall work impairment due to symptoms related to menstruation (WPAI 3). In addition, the difference in activity impairment outside work between cases and controls was measured as the percent activity impairment due to symptoms related to menstruation (WPAI 4). The protocol defined mild to normal menstruation as PBAC≤100 and VAS≤30). For the per protocol analysis the cutoff for HMB was set to PBAC 100. Thus, cases with PBAC≤100 and controls with PBAC>100 were excluded in this specific analysis.

All controls reporting heavy bleeding defined as PBAC>100, were contacted by the designated investigator and provided with adequate counseling. The hypothesis was that only a minority of healthy controls with self-perceived “mild to normal” menstruation would report a PBAC >100.

Statistical differences were considered significant at the 5% level; differences were assessed using Fisher's exact test for categorical data and Wilcoxon's rank-sum test for continuous data. Correlations between measures were measured using the Spearman rank coefficient.

A sample size calculation showed that a difference measured in hours of more than 9% between two groups could be shown by including 105 women in each group, at a significance level of 5% and power of 80%. We expected to recruit at least 150 women in each group to reach this number, to compensate for expected dropout due to ineligibility or non-response.

## Results

A total of 356 women (166 cases and 190 controls) were recruited from Jan 2015 to March 2017 at a total of nine sites in Sweden: Danderyds Hospital, Sabbatsbergs Hospital, The South Hospital and Södertälje Hospital in Stockholm, Helsingborg Hospital, The University Hospital in Örebro, Gallerians private practice in Jönköping, the Ingehammar private practice in Göteborg, the Wennerström private practice in Göteborg and the Hofte private practice in Helsingborg.

**Fig 1:**
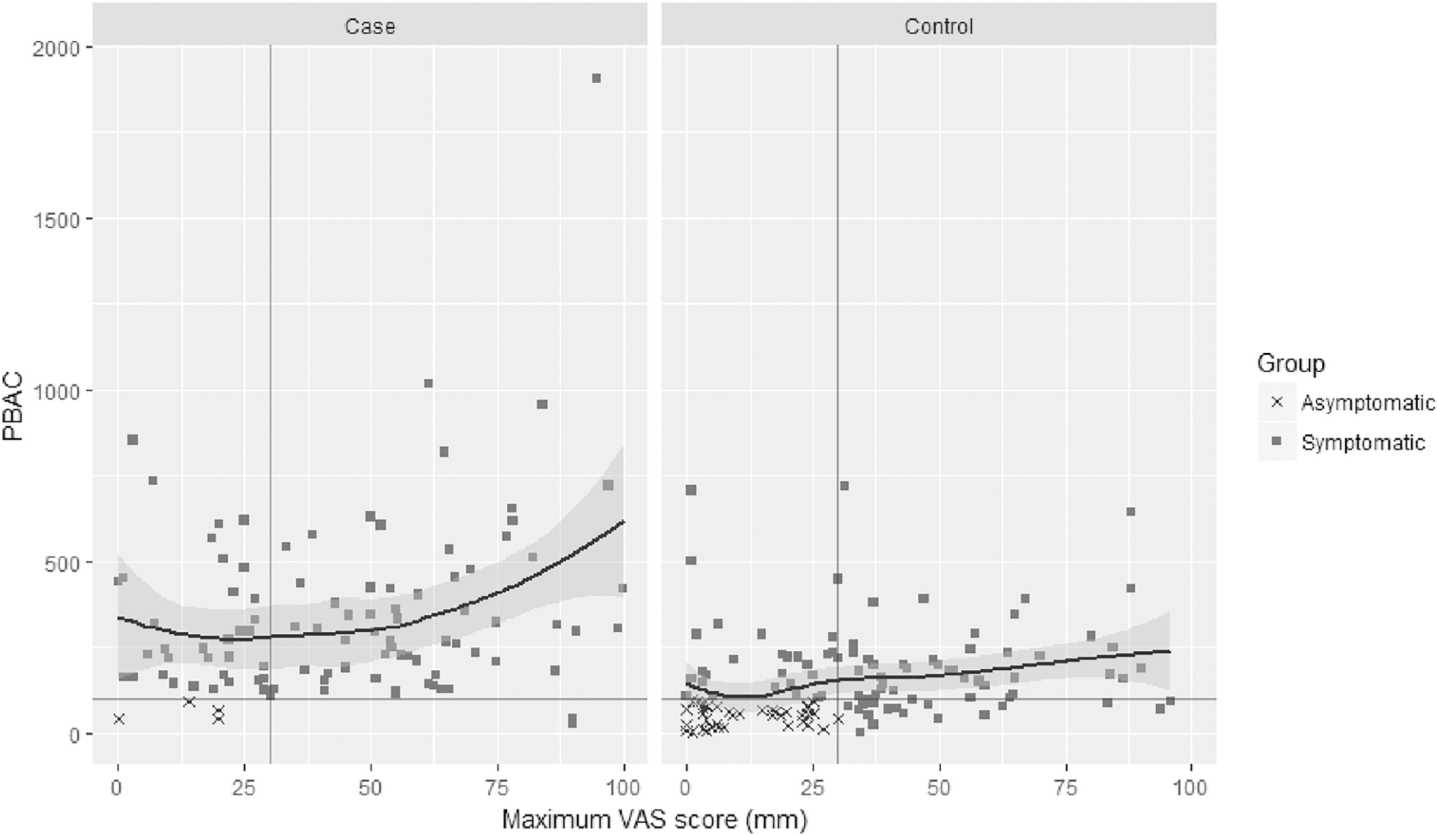
PBAC versus maximum VAS for all included subjects.

A total of 61 cases and 55 controls did not return any questionnaires. A total of 17 controls and 17 cases did not fulfill inclusion or exclusion criteria and were therefore excluded. For the final analysis 206 women remained whereof 91 cases and 118 controls, however some women failed to return one or more questionnaires. The distribution of all included cases and controls who reported PBAC and VAS are shown in Fig 1.

Out of 118 controls, 82 women (69.5%) were symptomatic according to the PBAC questionnaire (PBAC >100) and thus suffered from HMB, despite regarding themselves as having “mild to normal” menstruations. The results are therefore presented both for per protocol asymptomatic controls (reporting PBAC≤100) and for all self-reported asymptomatic controls (regardless of PBAC). The case group was significantly older than the control groups, had significantly heavier menstruations and reported higher levels of menstrual pain. In the case group, 3 of 91 (3%) women self-reported as having symptomatic despite PBAC≤100, this group was subsequently subdivided like the controls. The baseline characteristics of cases and controls are shown in Table 1.

**Table 1:** Baseline characteristics.

Group						
	Case (per protocol)	Control (per protocol)	p-value case vs control (per protocol)	Case (self reported)	Control (self reported)	p-value case vs control (self reported)
**Age (years)**						
N	88	36		91	118	
Mean (SD)	43.4 (5.2)	39.9 (5.3)	0.001	43.3 (5.3)	40.6 (5.8)	< 0.001
Median (IQR)	43.5 (40.8–47.0)	40.0 (36.0–43.2)		43.0 (40.5–47.0)	41.0 (36.0–45.0)	
Range	30.0–55.0	30.0–52.0		30.0–55.0	30.0–53.0	
**PBAC score**						
Mean (SD)	347.3 (261.4)	53.7 (26.1)	<0.001	338.1 (261.9)	151.2 (131.2)	< 0.001
Median (IQR)	282.5 (167.8–430.2)	57.0 (36.8–74.5)		270.0 (166.0–424.5)	107.0 (67.2–208.0)	
Range	26.0–1907.0	4.0–95.0		26.0–1907.0	2.0–718.0	
**VAS max**						
n	86	34		89	113	
Mean (SD)	45.1 (26.9)	12.3 (9.3)	<0.001	43.9 (27.2)	32.7 (25.0)	0.003
Median (IQR)	47.5 (23.2–63.8)	8.7 (4.0–19.9)		45.0 (21.9–63.0)	30.0 (10.4–43.8)	
Range	1.0–100.0	1.0–30.0		1.0–100.0	1.0–95.8	

Summary of patient demographics, PBAC-score, and VAS max by study group, p-values calculated by Wilcoxon sum-rank test. PBAC = pictorial blood assessment chart, VAS = visual analogue scale, SD = standard deviation, IQR = interquartile range

**Fig 2:**
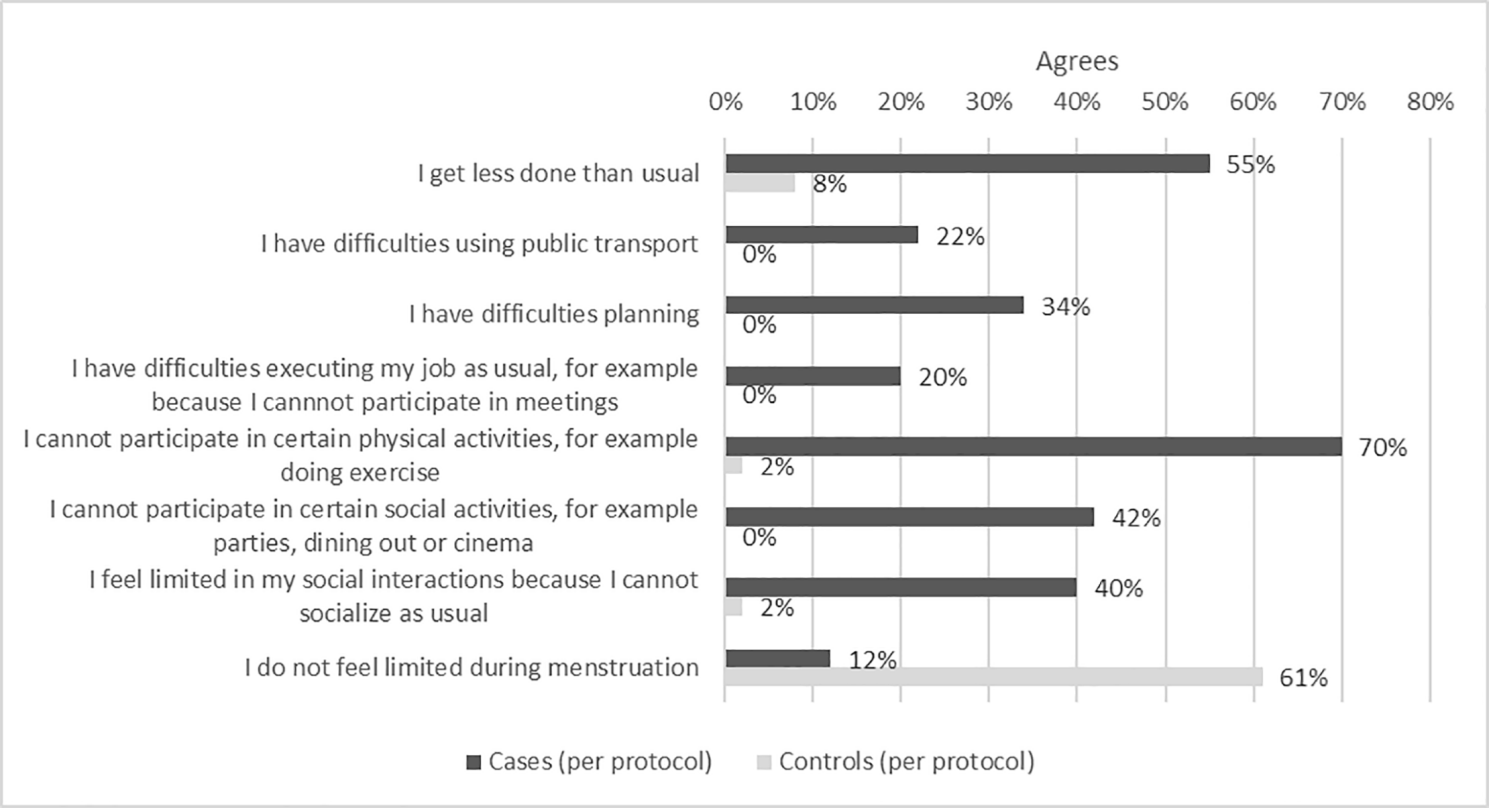
In what way do you feel limited during menstruation? Subjects were requested to agree or disagree with the above statements, the difference between the case and control group is significant for all individual items, p-values ≤ 0.001. P-values calculated by Fisher exact tests.

Results of primary and secondary outcomes are shown in Table 2. In the per-protocol analysis, cases reported significantly more absenteeism than controls, missing 7.6% work time due to menstrual problems compared to 0.2% (p = 0.003). In the self-reported groups cases missed 7.4% work time compared to 1.8% (p<0.001) work time missed by controls. There was also a significant difference between cases and controls in presenteeism, with cases reporting 43.8% impairment vs 12.1% among the controls (p<0.001). In the self-reported groups this difference remained (43.2 vs 22.6%; p<0.001). In the per-protocol asymptomatic control group only one woman missed time at work during menses, while at the same period 22/88 (25%) of cases missed time at work (data not shown). In addition, cases missed significantly more time at work due to other reasons than menstrual problems than both control groups (p = 0.04 for per protocol asymptomatic controls and p = 0.04 for self-reported asymptomatic controls, analyzed by Wilcoxon rank-sum test, data not shown). Result from the burden of illness questionnaire is shown in Fig 2. Cases were significantly more limited during menstruation than both control groups, in work related activities as well as in their spare time.

**Table 2:** Primary and secondary outcomes.

Group						
	Case (per protocol) (n = 88)	Control (per protocol) (n = 36)	p-value case vs control (per protocol)	Case (self reported) (n = 91)	Control (selfreported) (n = 118)	p-value case vs control (self reportedl)
**Primary outcome**						
**WPAI 1 absenteeism, percent work time missed due to menstrual problems**						
N	82	34		85	111	
Mean (SD)	7.6 (18.9)	0.2 (1.4)	0.003	7.4 (18.6)	1.8 (9.3)	< 0.001
Median (IQR)	0.0 (0.0–3.8)	0.0 (0.0–0.0)		0.0(0.0–3.1)	0.0 (0.0–0.0)	
Range	0.0–100.0	0.0–8.0		0.0–100.0	0.0–66.7	
Missing	6 (6.8%)	2 (5.6%)		6 (6.6%)	7 (5.9%)	
**Secodary outcomes**						
**WPAI 2 presenteeism, percent activity impairment while working due to menstrual problems**						
N	82	33		85	113	
Mean (SD)	43.8 (26.6)	12.1 (4.2)	< 0.001	43.2 (26.7)	22.6 (20.9)	< 0.001
Median (IQR)	40.0 (20.0–67.5)	10.0 (10.0–10.0)		40.0 (20.0–60.0)	10.0 (10.0–20.0)	
Range	10.0–100.0	10.0–20.0		10.0–100.0	10.0–100.0	
Missing	6 (6.8%)	3 (8.3%)		6 (6.6%)	5 (4.2%)	
**WPAI 3, percent overall work impairment due to menstrual problems**						
N	80	33		83	110	
Mean (SD)	46.3 (28.2)	12.3 (4.7)	< 0.001	45.6 (28.3)	22.5 (20.8)	< 0.001
Median (IQR)	40.0 (20.0–70.0)	10.0 (10.0–10.0)		40.0 (20.0–70.0)	10.0 (10.0–20.0)	
Range	10.0–100.0	10.0–26.4		10.0–100.0	10.0–100.0	
Missing	8 (9.0%)	3 (8.3%)		8 (8.8%)	8 (6.8%)	
**WPAI 4, percent activity impairment due to menstrual problems (time outside work)**						
N	85	34		88	114	
Mean (SD)	43.9 (26.4)	12.1 (4.1)	< 0.001	43.3 (26.5)	22.5 (20.8)	< 0.001
Median (IQR)	40.0 (20.0–70.0)	10.0 (10.0–10.0)		40.0 (20.0–62.5)	10.0 (10.0–20.0)	
Range	10.0–100.0	10.0–20.0		10.0–100.0	10.0–100.0	
Missing	3(3.4%)	2(5.5%)		3 (3.3%)	4(3.4%)	

Summary of WPAI outcomes, difference in work time missed and activity impairment, p-values calculated by Wilcoxon sum-rank test. SD = standard deviation, IQR = interquartile range

## Discussion

The morbidity due to leiomyoma which affects women of reproductive age is substantial. This has been acknowledged by research in the US, but only limited research has been published from European countries. To our knowledge there are no such studies from the Nordic countries, where health care is seldom financed by private or corporate health care insurance. The present study shows that women with leiomyoma are indeed significantly affected professionally and privately during menstruation. These women are missing a higher percentage of work time every menstrual period, and show higher activity impairment while at work than healthy controls. This is consistent with recent figures from Canada, which showed that women with symptomatic leiomyomas missed significantly more working hours per month and scored lower on the Uterine fibroids symptoms and health-related Quality of life (UFS-QOL) compared to matched controls without leiomyomas [12]. Furthermore, women with leiomyoma are absent from work more often than healthy controls due to other reasons. This has previously been addressed in other studies which have shown a considerably lower health-related quality of life and significantly higher depression severity score among women with leiomyoma [13, 14].

When recruiting controls for the present study, the main inclusion criteria was self-perceived “mild to normal menstruation”. Our hypothesis was that the majority of these women would report a PBAC≤100. Interestingly, close to 70% of the controls suffered from HMB measured as PBAC>100 [7]. The tendency to subjectively underestimate the menstrual blood loss has been previously described. In a pioneer population based study from Gothenburg, Sweden, 11% suffered from HMB with objective measurements. However 41% of these subjects considered their blood loss as moderate or scanty [6]. Even though the development and the access of hormonal contraceptives have improved over the last decades, the presence of HMB and/or misleading perceptions of MBL seem to hold true. In the present study, we underestimated the proportion of symptomatic women among controls. Interestingly, women underestimating their MBL, regarding themselves as having mild to normal menstruations in spite of PBAC>100, reported higher absence from work compared to controls with PBAC≤100 and VAS≤30. A higher cut off of PBAC has been proposed by other investigators and may have modified results [8].

There are limitations of the present study, including the relatively small sample size and the lack of specific data on the leiomyomas of the symptomatic women. Furthermore, we did not perform transvaginal ultrasound examinations of the healthy controls to rule out present leiomyomas and thus the risk of undiagnosed leiomyomas in this group is evident. The high proportion of controls with HMB, made us choose to subdivide the controls in two groups, in order to strengthen the transparency. We do not know the proportion of hormonal contraception use in either the cases or the control groups. Since the study design was focusing on symptomatology, knowledge of such treatment would not have changed the planned analysis of work impairment during menstruation. Symptoms related to menstruation were only recorded during one menstruation, however the day-to-day evaluation of PBAC and VAS, and the short recall period for WPAI (2–5 days) hopefully minimizes recall bias. The strengths of the study are the multicenter approach, and the objective to investigate base-line symptomatology of the participating women as most similar studies have investigated outcomes of different treatment modalities for leiomyoma.

Since medical and surgical treatment options for leiomyoma as well as HMB are many and increasing, today’s general practitioners and gynecologists have an important task to identify these patients, keep up with advances in the field, and choose appropriate treatments for each patient. A recent study from the Netherlands evaluated changing guidelines for general practitioners to treat HMB, showing that 40% of women seeking help for this condition received no treatment within 6 months of diagnosis, and only 18% were referred to a gynecologist [15]. Thus, there is evident room for improvement in the medical care of these women.

Our study of loss of productivity in women suffering from symptomatic leiomyomas and HMB in a Swedish setting shows that cases reported significantly more over all work impairment than controls which does not only constitute a distress for the women but also an expense for women and for society. We welcome further studies addressing these conditions and their impacts on work productivity, and urge health care providers to routinely assess women’s menstrual pattern, to timely detect symptoms which should lead to a referral to a gynecologist.

## Supporting information

S1 Dataset Contains raw data files. There are no direct identifiers.
(XLSX)
